# The complete chloroplast genome of Chinese medicine cultivar species of *Rehmannia glutinosa* (Orobanchaceae)

**DOI:** 10.1080/23802359.2020.1863163

**Published:** 2021-01-27

**Authors:** Zhi Xia, Cuicui Li, Saiwen Hu, Sheng Chen, Yuan Xu

**Affiliations:** a College of Agronomy, Henan Agricultural University, Zhengzhou, China; b College of Life Sciences, South China Agricultural University, Guangzhou, China; c Key Laboratory of Plant Resources Conservation and Sustainable Utilization, South China Botanical Garden, Chinese Academy of Sciences, Guangzhou, China

**Keywords:** *Rehmannia glutinosa* cultivar Wen 85-5, chloroplast genome, medicine cultivar species, Orobanchaceae

## Abstract

The cultivar of *Rehmannia glutinosa* (Orobanchaceae) is one of the four famous ‘Huai’ medicine cultivar species endemic to Henan Province in central China. In this study, we report the complete chloroplast (cp) genome of *R. glutinosa* cultivar Wen 85-5. The cp genome of *R. glutinosa* cultivar Wen 85-5 was 155,499 bp in length and contained a pair of inverted repeat regions (IR, 25,748 bp) separated by a small single copy (SSC, 17,600 bp) and a large single copy (LSC, 84,403 bp) region. Chloroplast genome sequences of two cultivar of *R. glutinosa* (Wen 85-5 and Jiwang 1) are identical to each other. The sequence of cp genome of *R. glutinosa* cultivar Wen 85-5 was 99.70% similar to the wild population of *R. glutinosa*. Some distinctive insert and deletion in *R. glutinosa* cultivar Wen 85-5 by comparison with wild population were reported. The maximum-likelihood phylogenetic analysis revealed that *R. glutinosa* cultivar Wen 85-5.was sister to the *R. glutinosa* cultivar Jiwang 1 (BS = 100%), and further clustering with *R. glutinosa* (BS = 100%). This result will be helpful for the conservation and breeding programs of the cultivar of *R. glutinosa*.

The genus *Rehmannia* Libosch. ex Fisch. & C. A. Mey. consists of six species endemic to China (Xia et al. 2009). Of these, *R. glutinosa* (Gaertn.) Libosch. ex Fisch. & C. A. Mey. is an important species in traditional Chinese medicine and also cultivated in Japan and Korea (Rix 1987). As one of the four famous ‘Huai’ medicines in Henan Province, *R. glutinosa* has a cultivation history in China for more than 1000 years. Although the complete chloroplast (cp) genome of six species in *Rehmannia* is reported by Zeng et al. (2017), the cultivar of *R. glutinosa* in its geo-authentic distribution is unknown. The cultivar of *R. glutinosa* was proved to derive from the wild population (Xia et al. 2018). Dense and hypertrophic leaves and expansion tuberous root are the main domestication trait of *R. glutinosa* cultivar Wen 85-5 (Figure 1(A)) which is different from wild population (Figure 1(B)). Modern pharmacological studies have demonstrated that the medicinal part of *R. glutinosa* mainly comes from the expansion tuberous root of cultivar of *R glutinosa*. In this study, we report the complete cp genome of *R. glutinosa* cultivar Wen 85-5, identify its distinctive insert and deletion, and infer phylogenetic relationships of this species and other taxa within *Rehmannia*.

**Figure 1.**
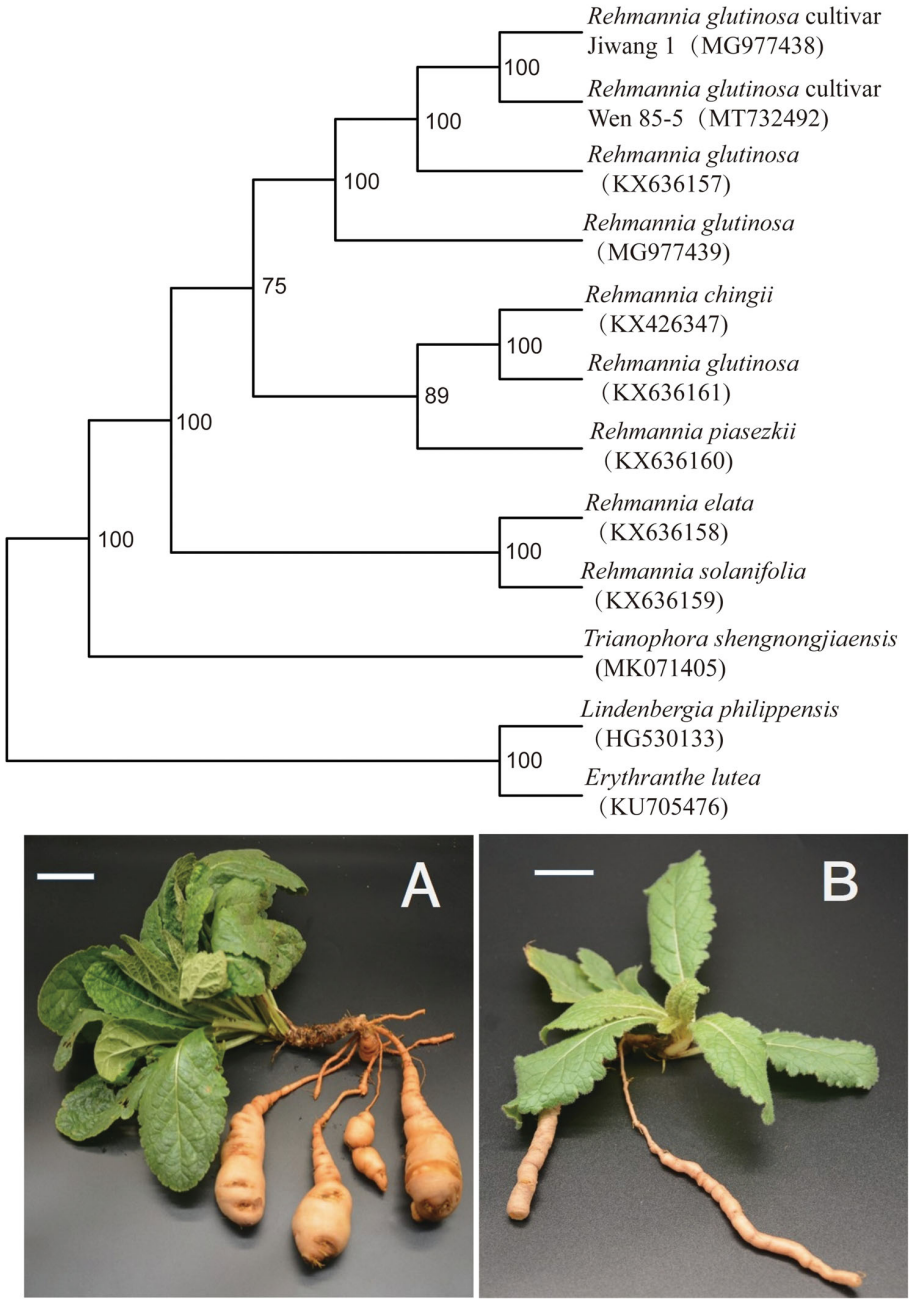
Maximum-likelihood phylogenetic tree based on 12 complete chloroplast genome sequences. The number on each node indicates the bootstrap value. (A) *Rehmannia glutinosa* cultivar Wen 85-5; (B) *Rehmannia glutinosa* wild population. Scale bars (A, B) 5 cm.

The fresh leaves of *R. glutinosa* cultivar Wen 85-5 were collected in Wuzhi County, Jiaozuo City, Henan Province, China (35°2′59″N, 113°13′35″E). The voucher specimens (voucher no.: XZ-2019-118) were deposited at the herbarium Henan Agricultural University (HEAC). The total genomic DNA was extracted following the CTAB method (Doyle and Doyle 1987). Then, the genomic library (paired-end, PE = 150 bp) was sequenced on an Illumine Hiseq 4000 platform at Beijing Genomics Institute (Shenzhen, China). Totally 2 Gb sequence reads were obtained and used to assemble the cp genome after filtering and trimming the low-quality reads and adaptor sequences. The complete cp genome assembly was executed on A5-miseq pipeline (Coil et al. 2015), with manual adjustment and annotation using Geneious version 11.0.3 (Kearse et al. 2012). *Rehmannia glutinosa* (GenBank accession number: KX636157) (Zeng et al. 2017) was used as reference plastid genome for assembling and annotation. The annotated GenBank files of complete cp sequence were submitted to GeneBank with the accession number MT732492.

The complete cp genome of *R. glutinosa* cultivar Wen 85-5 was 155,499 bp in length and contains a pair of inverted repeat (IRa and IRb) regions of 25,748 bp, the large single copy (LSC) region and small single copy (SSC) region with the lengths of 84,403 and 17,600 bp, respectively. A comparison of cp genome of *R. glutinosa* cultivar Wen 85-5 with *R. glutinosa* cultivar Jiwang 1 (Jeon et al. 2019) indicates that the sequences are identical to each other. The sequence of cp genome of *R. glutinosa* cultivar Wen 85-5 was 99.70% similar to the wild population of *R. glutinosa* (Zeng et al. 2017), 98.80% similar to *R. solanifolia*, 99.00% similar to *R. piasezkii*, 99.00% similar to *R. chingii*, 98.80% similar to *R. henryi*, and 99.10% similar to *R. elata*. Compared to the wild population of *R. glutinosa*, *R. glutinosa* cultivar Wen 85-5 has 791,218 and 86 bp insert in intergenic region of *trn*S-*trn*G, *rps*15-*ycf*1, *ycf*1-*ndh*F, *trn*P-*psa*J, and repeat regions, and 9,111,213 and 205 bp deletion in intergenic region of *psb*I-*trn*G, *ycf*4-cemA, *trn*T-*trn*L, *trn*F-*ndh*J, and *psb*A-*ycf*3.

A total of 12 complete cp genomes of *Rehmannia* and *Triaenophora* together with the obtained cp genome sequence in this study were utilized to clarify the phylogenetic position of *R. glutinosa* cultivar Wen 85-5, including *R. glutinosa* cultivar Jiwang 1 in Korea. Based on the result of Xia et al. (2019), we selected *Lindenbergia philippensis* and *Erythranthe lutea* as outgroups. All of the cp genome sequences were aligned with MAFFT (Katoh and Standley 2013). A maximum-likelihood analysis was performed with the RAxML software (Stamatakis 2014) using 1000 bootstrap replicates. The phylogenetic analysis reveals that *R. glutinosa* cultivar Wen 85-5.was sister to the *R. glutinosa* cultivar Jiwang 1 with maximum support (Figure 1). The two cultivars are clustered into *Rehmannia glutinosa* with maximum support (Figure 1). Our result supported that *R. glutinosa* cultivar Wen 85-5 and *R. glutinosa* cultivar Jiwang 1 may come from the domestication of *Rehmannia glutinosa*. The cp resource may be utilized for DNA barcoding, conservation genetics, and breeding of cultivar *Rehmannia glutinosa* in the future.

## Data Availability

The complete chloroplast genome sequence of *R. glutinosa* cultivar Wen 85-5 has been submitted to the GenBank (https://www.ncbi.nlm.nih.gov/genbank/), and the accession number is MT732492. This sequence has been released in NCBI. The raw sequencing data have been submitted to NCBI SRA database, here is the accession link: https://www.ncbi.nlm.nih.gov/sra/?term=PRJNA665347.
